# Gender and cultural bias in student evaluations: Why representation matters

**DOI:** 10.1371/journal.pone.0209749

**Published:** 2019-02-13

**Authors:** Y. Fan, L. J. Shepherd, E. Slavich, D. Waters, M. Stone, R. Abel, E. L. Johnston

**Affiliations:** 1 School of Mathematics and Statistics, UNSW, Sydney, Australia; 2 Department of Government and International Relations, The University of Sydney, Sydney, Australia; 3 Division of Academic, UNSW Sydney, Australia; 4 Division of Research, UNSW Sydney, Australia; 5 School of Biological, Earth and Environmental Sciences, UNSW Sydney, Australia; University of Indianapolis, UNITED STATES

## Abstract

Gendered and racial inequalities persist in even the most progressive of workplaces. There is increasing evidence to suggest that all aspects of employment, from hiring to performance evaluation to promotion, are affected by gender and cultural background. In higher education, bias in performance evaluation has been posited as one of the reasons why few women make it to the upper echelons of the academic hierarchy. With unprecedented access to institution-wide student survey data from a large public university in Australia, we investigated the role of conscious or unconscious bias in terms of gender and cultural background. We found potential bias against women and teachers with non-English speaking backgrounds. Our findings suggest that bias may decrease with better representation of minority groups in the university workforce. Our findings have implications for society beyond the academy, as over 40% of the Australian population now go to university, and graduates may carry these biases with them into the workforce.

## Introduction

Using student evaluations of teaching (SET) as a tool to assess teaching quality has become a contentious issue. Some scholars ([24]) argue that these surveys do not measure teaching effectiveness and should only be used to monitor student experience. Yet many academic institutions require the reporting of SET results as a routine component of performance enhancement and promotion. A number of recent influential studies ([11], [6], [19], [7]) have found evidence of gender bias in university teaching evaluations. Indeed, several studies have found that gender, ethnicity and the instructor’s age matter ([2], [9], [4] [26], [27]). While the literature on teaching evaluations is rich, most studies either rely on case studies, or small sample sizes.

Recently for example, a study of around 20,000 student evaluations over the period 2009-2013 from the school of Business and Economics, [11] at the University of Maastricht in the Netherlands, found that, on average, female instructors received a score 37 percentage points lower than male instructors. The bias is driven by male students, and is worst for junior female instructors. They also found the bias to be more obvious in courses which contain more mathematics. Another study from a French university analysed over 22,000 online evaluations over a 5 year period for students in social sciences ([6]). They found that male students express a bias in favour of male professors, and that men are perceived to be more knowledgeable and to have stronger leadership skills. Finally, a US study conducted an experiment whereby the instructors of an online course operated under two differently gendered avatars ([19]). This research found that students rated the male avatar significantly higher than the female avatar, regardless of the instructor’s actual gender, but the study was based on a sample size of 43 students assigned to 4 different instructors.

There is very little research on the effect of culture or race on SET scores. Some authors ([10], [13]) have studied course evaluation scores between Hispanic and Asian-American faculty compared to White faulty. However, the sample size used for the analyses was too small to draw any conclusions. Other studies have also been carried out, using surveys or interviews ([23], [14]). In the Australian context, public conversations have been focussed primarily on gender equality. One recent report found that Asian Australian academics perceive their heritage as a disadvantage in the workplace ([22]), whilst others have argued there is resistance in opening such debates ([15]).

This study is based on SET and course satisfaction data collected at a leading Australian university, which consistently collected student evaluations of courses and teaching over a long period of time. We refer to these data throughout as “SET data”. The dataset is comprised of 523,703 individual student surveys, across 5 different faculties and over a seven year period 2010-2016. There were 2,392 unique courses and 3,123 individual teachers in the dataset. The university has a high international student population, (comprising 34% of the surveys), primarily from the Asia-Pacific region, and a diverse international cultural background in the teaching staff (38% of the classification). See Table 1 for a break down of the demographics.

**Table 1:** Breakdown of demographics from the SET dataset by faculty. Across the rows are: total number of individual student surveys; total number of unique courses; number of female teachers with non-English and English speaking background; number of male teachers with non-English (NE) and English (E) speaking background; and the number of female and male international (I) and local (L) students.

	Bus	Sci	Med	Eng	Arts	Totals
Total Surveys	165533	111728	24052	60699	161691	523703
No. Courses	439	395	123	537	898	2392
Female Teachers (E)	113	116	111	25	296	661
Male Teachers (E)	220	163	99	114	204	800
Female Teachers (NE)	113	56	45	26	128	368
Male Teachers (NE)	223	90	32	115	76	536
Female Teachers (unclassified)	55	26	35	14	220	350
Male Teachers (unclassified)	114	67	34	65	128	408
Female Student (L)	6011	5887	2397	1423	9856	25574
Male Student (L)	7965	6873	1597	4666	4474	25575
Female Student (I)	6221	1891	667	1503	3345	13627
Male Student (I)	4667	2468	507	4122	1371	13135

This study differs from all previous studies in several ways. First, our study is by far the largest data study and the only institution-wide study of SET; second, we look at evidence for potential cultural bias and the interplay between gender and cultural bias in a way that has never been considered (We are, of course, mindful that ‘culture’ is a complex and contested concept. We use the term ‘cultural bias’ to capture the combination of biases related to language background, embodiment or presentation of (presumed) racial/ethnic identity, and beliefs or conventions particular to a given cultural context. In our dataset, ‘language spoken at home’ is the relevant variable.); finally we use a random effects model to appropriately account for “course” and “teacher” effects in a statistically rigorous analysis.

## Methods

This research was approved by the UNSW Human Research Ethics Advisory Panel (HREAP), HC17088.

### Data collection

The university has a mature data warehouse that has been developed using the Kimball method of data warehousing ([18]). The method models individual business processes subject by subject to form an enterprise warehouse. Integration between subjects is achieved by adhering to a data warehouse bus matrix which captures the relationships between the business processes and the core descriptive dimensions. This enables subject-oriented data marts to be built over time and be assembled to produce an Enterprise Data Warehouse ([25]). The resulting integrated data warehouse is optimized for reporting and analytics ([18]). This data is used for many of the university’s decision support processes and has been cleansed, tested and utilized for decision making for seven years.

Seven of the business processes that have been modelled have been used to prepare the data for the analysis work. These processes are program creation, course creation, enrolment in programs, enrolment in courses, grades in courses, accumulative weighted average mark (WAM) in a semester and course survey. As part of the ethics approval on this project we separated the data preparation and engineering from the data analysis. The data was prepared and anonymized to protect the identity of the students doing the survey and the teachers who are the subject of the survey. The anonymized data was then handed over for analysis.

The data set is itemised by students enrolled in courses. Each semester students enrol in courses and at the end of each semester students are asked to participate in a survey about their experience in the course. The survey is voluntary, and anonymous, students are reassured that they cannot be identified and penalised for their comments.

In the data set, attributes of the courses and programs were retained such as faculty (The term ‘faculty’ is used here to refer to the administrative unit of the university (there are eight faculties at the university in question, including Arts and Social Sciences, Business, Science and so on), not to be confused with the teacher/professor.), school, re-identified unique code, re-identified unique name and the field of education. The Field of Education is the Australian Bureau of Statistic’s Australian Standard Classification of Education (ASCED) ([3]). Teacher demographics were included to aid analysis. This included re-identified teacher identifier, gender, age at survey time, Australian residency information, citizenship information, language spoken at home, indigenous status and salary grade (Casual Tutorial, Casual Lecturer, Associate Lecturer, Lecturer, Senior Lecturer, Associate Professor or Professor). In the Australian system, a casual tutor and casual lecturer may or may not hold PhDs. associate lecturers are often temporary lecturers with a PhD, lecturer is equivalent to tenure track assistant professors in the North American system, and senior lecturer/associate professor is equivalent to the associate professor then professor to professor in the North American equivalent.

Student demographics to aid analysis including re-identified respondent identifier, WAM at survey time, gender of the student, age at survey time, Australian residency information, citizenship information, language spoken at home, indigenous status, grade for specific course being surveyed, student load for semester of the survey (is the student part time or full time). The student is asked for demographic information on the survey and this is also included. This data includes, gender as stated in survey response, mode of study as stated in survey response, residency as stated in survey response.

The university has been performing Course and Teaching Evaluation and Improvement (CATEI) surveys in one form or another since the late 1990s and moved online in the late 2000s. The survey data used for this analysis are from 2010-2016, and included four questionnaire forms:

- Form A (Course Evaluation) which was used to evaluate a course;
- Form B (Large Group Teaching Evaluation) which was used to evaluate course lecturers;
- Form C (Small Group Teaching Evaluation) which was used to evaluate tutors or lab demonstrators; and
- Form D (Studio/Design Based Teaching) which was used to evaluate tutors or studios with smaller number of students.

The Likert questions (from a scale of 1 to 6, “strongly disagree, disagree, moderately disagree, moderately agree, agree, strongly agree”) on each form consisted of up to ten questions, eight standard questions in the case of Form A and two text questions. Forms B, C and D comprised of seven standard questions and up to two text questions. This analysis focus on the last question:

- Form A (Course Evaluation) Overall, I was satisfied with the quality of this course.
- Form B (Large Group Teaching Evaluation) Overall, I was satisfied with the quality of this lecturer’s teaching.
- Form C (Small Group Teaching Evaluation) Overall, I was satisfied with the quality of this facilitator’s / tutor’s teaching.
- Form D (Studio teaching Evaluation) Overall, I was satisfied with the quality of this facilitator’s / tutor’s teaching.

Classes at this university were predominantly conducted in the traditional way during the survey period, i.e., face to face lectures and tutorials or labs where students are expected to attend. Large groups of lectures can have up to two to three hundred students, while typical tutorials and lab groups are under 30 students. Our focus on the final survey question is based on the fact that this is the question used by management as performance indicators for promotion and other purposes.

### Statistical analysis

Individual student evaluations scores (for a particular teacher from a particular course) are measured on a Likert scale (1, …, 6), indicating “strongly disagree, disagree, moderately disagree, moderately agree, agree, strongly agree”. Together with the score, we also have information on a variety of student, teacher and course specific variables. An ordinal regression model is appropriate for this type of response, since scores are ordered categorical data ([1]).

Since the data we analyse here is observational, the unequal number of times that a course or a teacher is surveyed can lead to biased results. To account for this we use a mixed model with two random effects terms to account for individual course effects, and individual teacher effects, these two terms will also pick up individual specific effects not otherwise accounted for in the model. The number of students providing multiple surveys to the same teacher is few, therefore we treat the responses as conditionally independent.

A large number of studies have produced mixed conclusions about which student or teacher characteristics influence SET results, but most of these are based on small samples or case studies ([5]). We include in our fixed effects most of the frequently studied variables, including student semester average mark (WAM); student cultural background: as indicated by residency status of student; gender of student; total number of students in the course; course type (postgraduate or undergraduate); gender of teacher; and cultural background of teacher (English or non-English background). Around one third of teachers had missing information in the database that contained language/cultural background of the teacher information- in these cases we flagged them to be English speaking if they were born in a predominantly English speaking country (Australia, New Zealand, United Kingdom, United States, South Africa) and non English speaking if they were born elsewhere. Where country of birth and language spoken at home were both missing we flagged the cultural background as missing, unless the citizenship status was a non Australian class- in which case we flagged the cultural background as non English speaking. Overall, 24% of teachers were flagged with missing cultural background. Since the interplay between student attributes and teacher attributes are complicated, we include four further interaction terms between: teacher gender and student cultural background; teacher gender and student gender; teacher and student cultural backgrounds; and teacher cultural background and student gender. All terms are treated as linear here, based on findings from the relevant literature ([5]).

We fit a cumulative logit link model of the form

$$\begin{array}{c} \log(\frac{P(y_{ict}\leq j)}{1-P(y_{ict}\leq j)})=\theta_{j}-x_{i}^{T}\beta -\alpha_{c}-\alpha_{t} \end{array}$$ (1)

where *j* = 1, …, 6 refer to the response levels, *P*(*y*_*ict*_ ≤ *j*) is the probability of student *i* from course *c* taught by teacher *t* giving a score less than or equal to level *j*, given **x**_*i*_ = (*x*_1*i*_, …, *x_pi_*) the vector of fixed effect measurements and *α_c_* and *α_t_* are the vector of random effects coefficients. The vector *β* = (*β*_1_, …, *β_p_*) is the vector of fixed effects coefficients. The model was fitted separately to each faculty using the *ordinal* package which uses a maximum likelihood approach in *R* [21].

### Interpretation of fixed effects parameters

The effect of gender or culture can be studied through the fixed effect coefficient for the particular effect. For instance, if we are interested in the gender effect, the covariate for gender **x**_*k*_ takes values 0 or 1 indicating female and male. Then Eq 1 for women becomes $log\left(\frac{P(y_{ict}\leq j)}{1-P(y_{ict}\leq j)}\right)=\theta_{j}-x_{i,-k}^{T}\beta_{-k}-\alpha_{c}-\alpha_{t}$, i.e., the *β*_*k*_ term disappears from the equation for women. Then because **x**_*k*_ takes the value 1 for men, *β*_*k*_ stays in the equation for male teachers. Taking the difference between the equation for female and male teachers, we get

$$\begin{array}{c} \log\left(\frac{\;\text{odds}\;\text{females}}{\;\text{odds}\;\text{males}}\right)=\beta_{k} \end{array}$$ (2)

where odds females is defined as *p*_*j*_/(1 − *p*_*j*_), *p*_*j*_ = *P*(*y*_*ict*_ > *j*) for women, and the odds males is defined as *q*_*j*_/(1 − *q*_*j*_), *q*_*j*_ = *P*(*y*_*ict*_ > *j*) for men.

As the model included interaction terms with student gender *(and cultural background)*, we calculated the odds ratios separately for each strata of students (male and female students, *and local and international students)*. The 95% confidence intervals were calculated for the odds ratio. The standard error of the log-odds ratio followed naturally from the inverse of the Hessian, a by product of the model fit. Then *OR* ± 1.96*e*^*se*(log(*OR*))^.

### Subset analysis

In order to gain a sense of relative contribution of gender and culture to factors that actually measure improvements in teaching effectiveness, we created a new variable that indicates if the course is being taught at least once before by the instructor. Typically, instructors’ scores improve by a large amount once they have taught the course once, and have had feedback on the course. To do this, we use data only from 2012 onwards, and only data on teachers appointed at the lecturer or senior lecturer level. These staff conduct the bulk of academic teaching, and there is less variability amongst this cohort than amongst the casual teaching staff. We created a flag to indicate whether the instructor has not taught the course in the last 3 years. We assume if the instructor has not taught the course in the last 3 years, they can be considered as teaching the course for the first time. We fit a model as above with random effects to account for SET scores clustered on teacher and course, and fixed effects terms student WAM, student cultural background, gender of student, total number of students in course, course type, gender of teacher, cultural background of teacher, and whether the teacher has experience teaching the course (we did not fit interactions here as the dataset was reduced in size).

### Model assessment

To assess the ability of the ordinal regression at classifying scores, for each *j* = 1, …, 5 we took the estimated probability that the SET score is less than or equal to *j* (i.e. $\hat{P}\left(y_{ict}\leq j\right)$) and compared that to a binary indicator for whether the observed SET score was less than or equal to *j* (i.e. $1\{Y_{ict}^{obs}\leq j\}$). We calculated the Area under the Receiver Operating Curve (AUC), which assesses how well $\hat{P}\left(y_{ict}\leq j\right)$ is able to discriminate $1\{Y_{ict}^{obs}\leq j\}$. Generally AUC’s between 0.7-0.8 are considered fair, 0.8-0.9, good and 0.9-1 excellent ([17]).

To assess uncertainty in the AUC from a mixed model, accounting for the design clustered on teachers and courses, we conduct a clustered bootstrap ([20]). That is, we sample course-teacher units in each of Nboot = 500 resamples. Letting (c*, t*) be the resampled indices, the standard error of AUC was estimated from sd(AUC($\left(\hat{P}\left(y_{ic^{*}t^{*}}\leq j\right),1\left\{Y_{ic^{*}t^{*}}^{obs}\leq j\right\}\right)$), and 95% confidence interval limits for the AUC were then estimated as AUC ±*z* se(AUC), where z = 1.96, a common large sample approximation for AUC (e.g. [12]).

## Results

### Gender and cultural effect

We found significant association between SET scores and gender, as well as culture, where the variable indicating whether the teacher has English or non-English background as defined in the Statistical Analysis section, is used as a proxy for culture. About 38% of the university’s teaching staff do not have English speaking background, and this population is racially diverse, comprising of people from all continents, but predominantly from Europe, Asia and the Americas.

**Fig 1:**
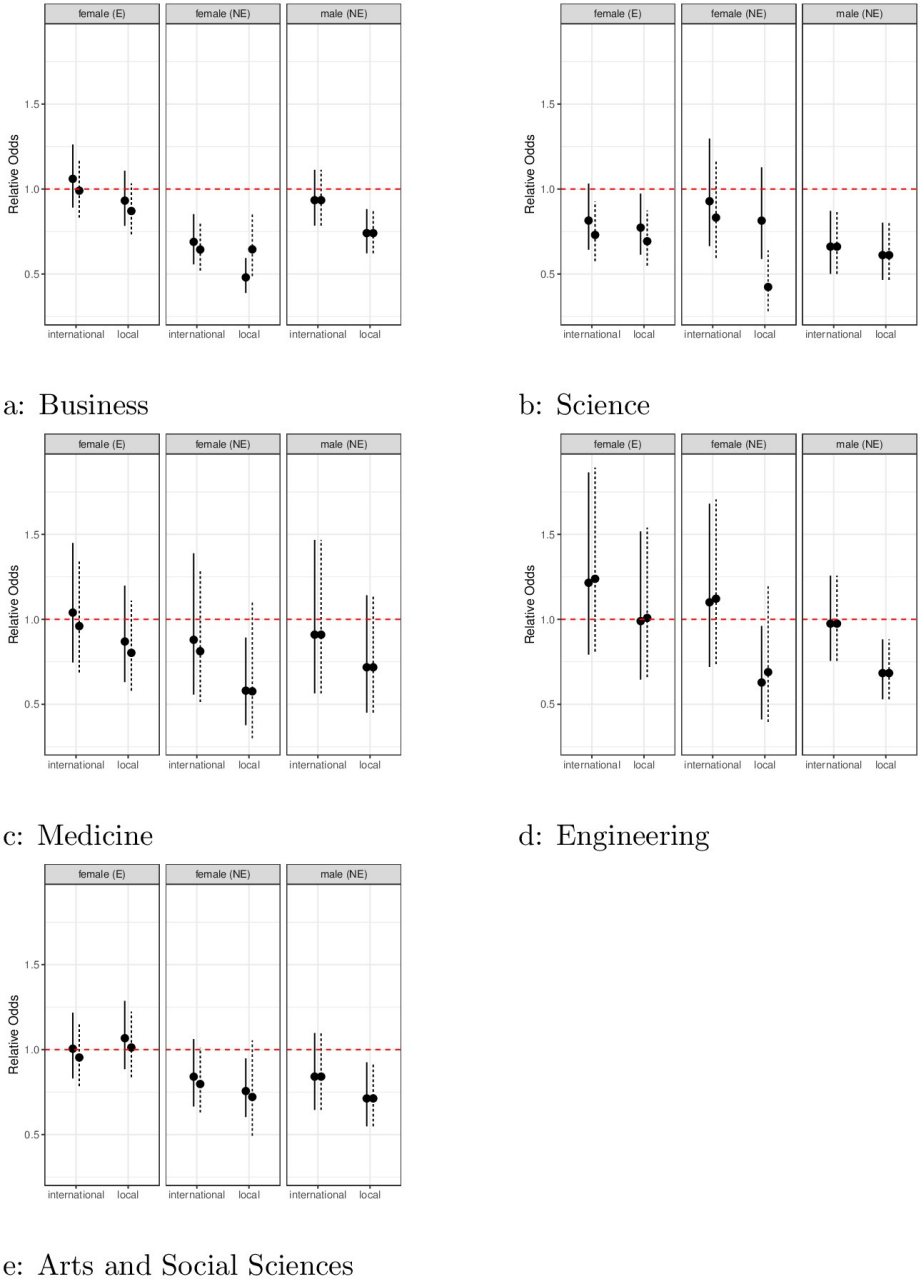
Effect of gender from teaching evaluations. Points below the line at one indicate bias against groups (English (E) and non-English (NE) speaking females, and non-English (NE) speaking males) across male and female local and international students. Where solid lines (female students), and dotted lines (male students) indicate 95% confidence interval. If vertical lines do not intersect the line at 1, this indicates differences are statistically significant.

Across five different faculties, the gender and cultural effects generally have a negative impact on the SET scores of women and teachers of non-English-speaking backgrounds across almost all faculties and subgroups, see Fig 1. Even when other factors such as individual course variation, individual teacher variation, student average score (WAM), course type and so on are accounted for, gender and culture are found to be statistically significant in some faculties, especially in Science and Business.

It is informative to look at the most affected group: female instructors from non-English speaking backgrounds. This is a substantive group, comprising around 38% of the female teaching workforce. In all faculties, a statistically significant effect against them is observed, with the effect stronger among local students. In the worst case, the science faculty, the relative odds of female non-English speakers getting a higher SET score is around 42% from local male students when compared to men from English speaking backgrounds. In other words, the odds of a male English speaker getting a higher score is more than twice that of a female non-English speaker. The results in Business were around 55% (0.48,0.65), or 1.82 times, whilst Engineering and Medicine faculties are a little better, at around 62% (0.58,0.58, 0.63,0.69) from Medicine and Engineering respectively, or 1.61 times, see Table 2 for exact numbers.

**Table 2:** Relative odds or effect size for different teacher/student populations. Columns indicate student attribute and rows indicate teacher attribute. Confidence intervals are given in brackets, and significant (at 5% level) terms are highlighted in bold font. Confidence intervals not including the value 1 indicates significance.

Instructor	Faculty	Int_Female	Int_Male	Local_Female	Local_Male
Female (E)	Bus	1.06(0.89,1.26)	0.99(0.83,1.18)	0.93(0.78,1.11)	0.87(0.73,1.03)
Female (NE)	Bus	**0.69(0.56,0.85)**	**0.64(0.52,0.8)**	**0.48(0.39,0.59)**	**0.65(0.49,0.85)**
Male (NE)	Bus	0.93(0.79,1.11)	0.93(0.79,1.11)	**0.74(0.62,0.88)**	**0.74(0.62,0.88)**
Female (E)	Sci	0.82(0.64,1.03)	**0.73(0.58,0.93)**	**0.77(0.61,0.97)**	**0.69(0.55,0.87)**
Female (NE)	Sci	0.93(0.66,1.3)	0.83(0.6,1.16)	0.81(0.59,1.13)	**0.42(0.28,0.64)**
Male (NE)	Sci	**0.66(0.5,0.87)**	**0.66(0.5,0.87)**	**0.61(0.47,0.8)**	**0.61(0.47,0.8)**
Female (E)	Med	1.04(0.75,1.45)	0.96(0.69,1.34)	0.87(0.63,1.2)	0.8(0.58,1.11)
Female (NE)	Med	0.88(0.56,1.39)	0.81(0.51,1.28)	**0.58(0.38,0.89)**	0.58(0.3,1.11)
Male (NE)	Med	0.91(0.56,1.47)	0.91(0.56,1.47)	0.72(0.45,1.14)	0.72(0.45,1.14)
Female (E)	Eng	1.22(0.79,1.86)	1.24(0.81,1.89)	0.99(0.65,1.52)	1.01(0.66,1.54)
Female (NE)	Eng	1.1(0.72,1.68)	1.12(0.74,1.71)	**0.63(0.41,0.96)**	0.69(0.4,1.2)
Male (NE)	Eng	0.97(0.76,1.26)	0.97(0.76,1.26)	**0.68(0.53,0.88)**	**0.68(0.53,0.88)**
Female (E)	Arts	1.01(0.83,1.22)	0.95(0.79,1.16)	1.07(0.89,1.29)	1.01(0.84,1.22)
Female (NE)	Arts	0.84(0.67,1.06)	0.8(0.63,1.01)	**0.76(0.6,0.95)**	0.72(0.49,1.05)
Male (NE)	Arts	0.84(0.65,1.1)	0.84(0.65,1.1)	**0.71(0.55,0.92)**	**0.71(0.55,0.92)**

Ignoring any cultural effects, and looking only at the cohort of female English speakers, we find significant effect against female instructors primarily in the Science faculty. Where the odds of female English speaking teachers getting higher scores is around 80% (from female students) and 70% (from male students), compared with their male counterparts. That is, men have 1.25 times the odds of women getting higher scores from female students, and 1.43 times from male students.

In Arts and Social Sciences, although we find no statistically significant gender effect against women in the English speaking cohort, significant cultural effect is observed against both male and female non-English speaking teachers, when evaluated by local students. The faculties of Engineering and Medicine demonstrate similar behaviours, with the only significant bias found against the non-English speaking female cohort when evaluated by local students. In the case of Engineering, a significant effect against non-English speaking male teachers by local students is also observed. Results from these latter two faculties have large standard errors compared to the other faculties, suggesting a high variability in the SET scores, reflective of the relatively small numbers of surveys from these two faculties (60,699 and 24,052).

To investigate potential bias in different student cohort, the model including interaction between student program (undergraduate or postgraduate) and gender did not show statistical significance. This suggests that the biases we find here are most likely ingrained in our culture rather than specific to the university environment, since there is no evidence that bias changes between the undergraduate degree and postgraduate degree. This also suggests that students will likely carry these biases with them when they graduate.

**Fig 2:**
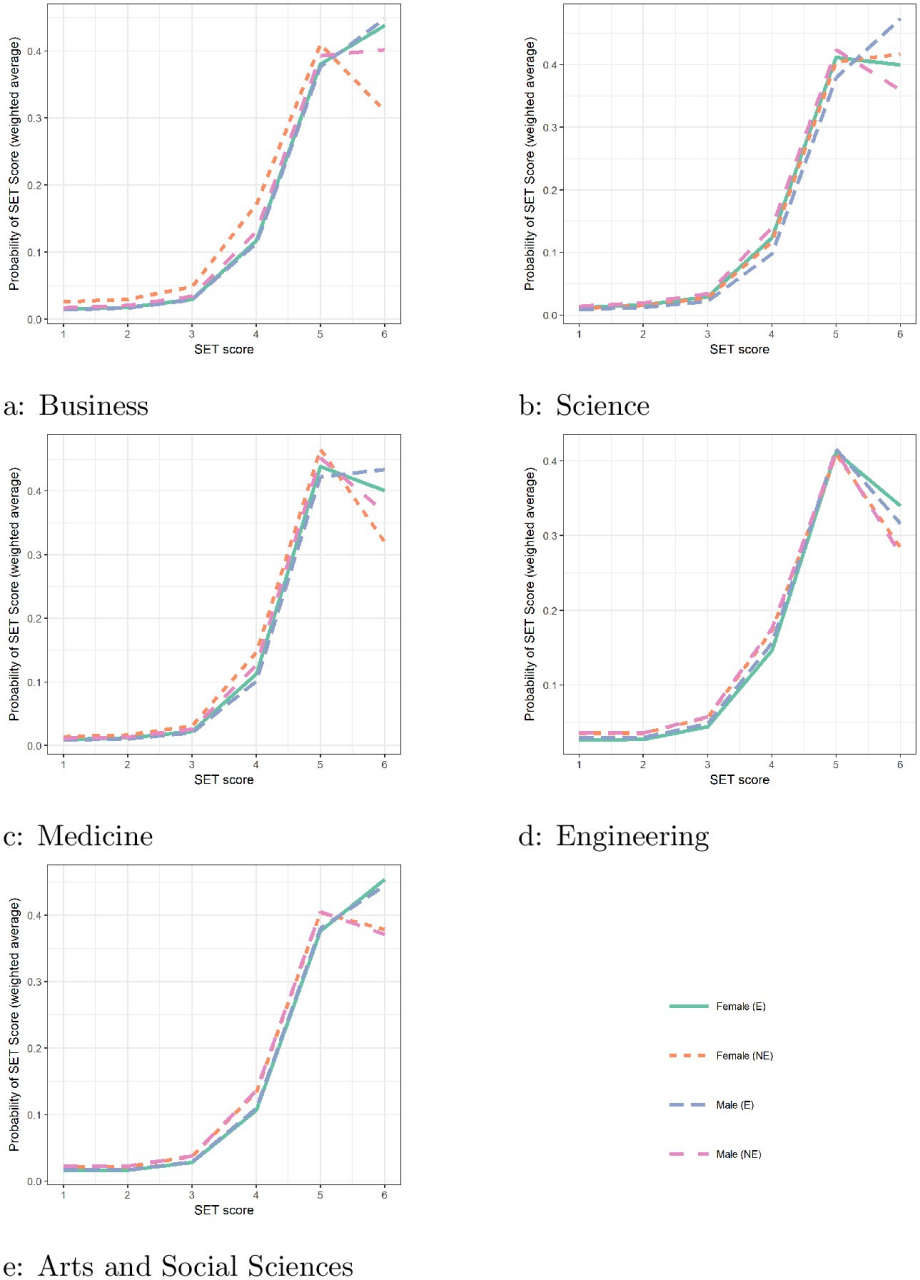
Estimated average probability of scoring *j* = 1, …, 6, *P*(*Y* = *j*). The scores are plotted on the axis and the corresponding *P*(*Y* = *j*) on axis. Different line types correspond to different gender and culture groups.

Fig 2 shows the estimated probabilities for SET scores (1, …, 6) for different faculties. In all but Engineering, male English speaking teachers have the highest probability of getting the highest possible grade at 6 (out of 6 possible scores): this probability increases almost linearly over lower scores. It is interesting to note that in Engineering, the only faculty where male teachers with English speaking background do not have the highest probability of scoring the maximum point of 6, the differences between the gender culture groups is small. The probabilities are around 0.3, compared to the much higher average of around 0.4 for the other faculties.

Around 80% of the scores are given at either 5 or 6 and our results suggest that bias comes in at this top level, between “agree” and “strongly agree”. Students appear to be more at ease with giving the highest scores to the dominant group (male with English background) particularly in Science. It should be noted that it is also difficult to numerically quantify how big the difference really is, as the numbers 5 and 6 do not necessarily reflect the magnitude of the difference between “agree” and “strongly agree” [24] (which is qualitative), compared with “moderately agree” and “agree”, in both cases, the numerical difference is 1.

### Comparisons with course evaluations

**Fig 3:**
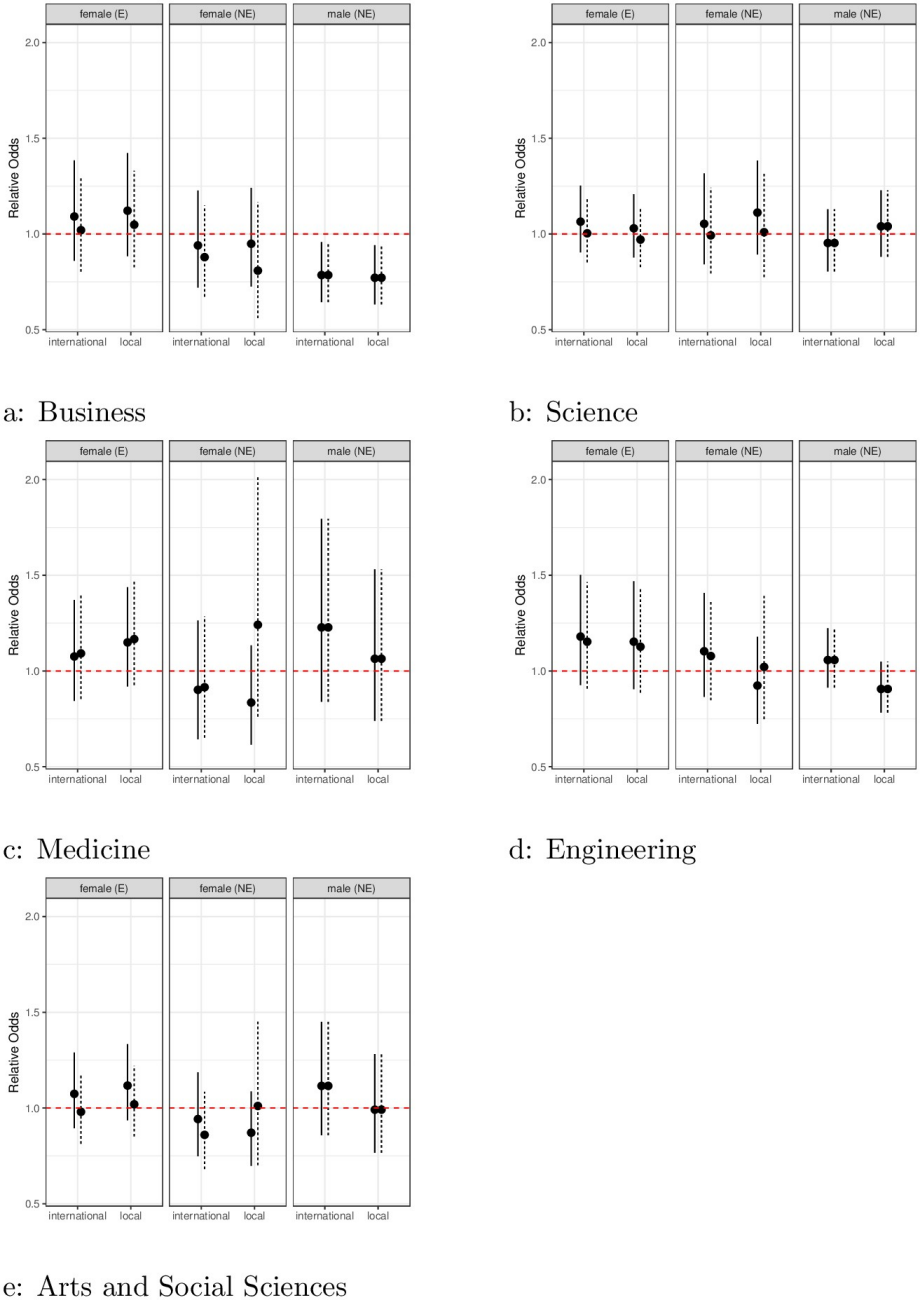
Gender effect from course evaluations. Points below the line at one indicate bias against groups (English (E) and non-English (NE) speaking females, and non-English (NE) speaking males) across male and female local and international students. Where solid lines (female students), and dotted lines (male students) indicate 95% confidence interval. If vertical lines do not intersect the line at 1, this indicates differences are statistically significant.

Unlike SET surveys, questions on course evaluations do not ask the students to evaluate the *teacher*, but the quality of the *course*, in this case responding to the statement “Overall I was satisfied with the quality of the course”. Since typically a course receives both teaching and course evaluations, it is instructive to look at the effects of gender and culture on course evaluations as a comparison with teaching evaluations. The results from fitting the same statistical model to course evaluations data are shown in Fig 3 (the equivalent of Fig 1 for teaching). It is interesting to note here that the strong gender and culture effects seen in the teaching evaluations are no longer present in the course evaluations. For instance, in Science, women had 70% odds of getting a better score in teaching evaluations, this number goes up to around 100% in course evaluations. These results suggest that biases creep in when students evaluate the person, not the course.

### Interplay between student and teacher attributes

The interplay between gender and culture is complicated. For instance, in Science, the cultural effect appears to override gender effect: with men from non-English speaking backgrounds not getting higher scores despite the fact that they are male. Students’ own cultural background does not play a prominent role. However, male students give lower scores to female teachers regardless of the cultural backgrounds of either student or instructor. Across other faculties, local students generally rank both female and those with non-English speaking backgrounds lower than international students rank them. Table 3 shows significant interaction effects between gender of the student, gender of teacher, cultural background of students and cultural background of teacher. For instance, in the business school, under the first column, there is significant interaction between student culture and teacher gender, this might mean that local students prefer males while international students favour females. The results here give support to the argument that we unconsciously preference people who are more similar to ourselves, regardless of whether that similarity arises through gender or culture.

**Table 3:** P-values for the effect size of the interaction terms, for different faculties. Significant terms (at 5% level) are highlighted in bold font.

Interaction	Bus	Sci	Med	Eng	Arts
Student Culture: Teacher Gender	**0.00**	0.11	**0.01**	**0.00**	**0.03**
Student Culture: Teacher Culture	**0.00**	**0.03**	**0.00**	**0.00**	**0.00**
Student Gender: Teacher Gender	**0.00**	**0.00**	0.13	0.71	**0.02**
Teacher Gender: Teacher Culture	**0.01**	**0.01**	0.82	0.80	0.97

### The effect of representation

Our results suggest that where there are larger proportions of female teachers, such as in the Arts and Social Sciences, there is less gender bias in student evaluations of teaching. In Science, where the largest proportion of staff are male English speakers, we have observed stronger biases against the minority groups.

**Fig 4:**
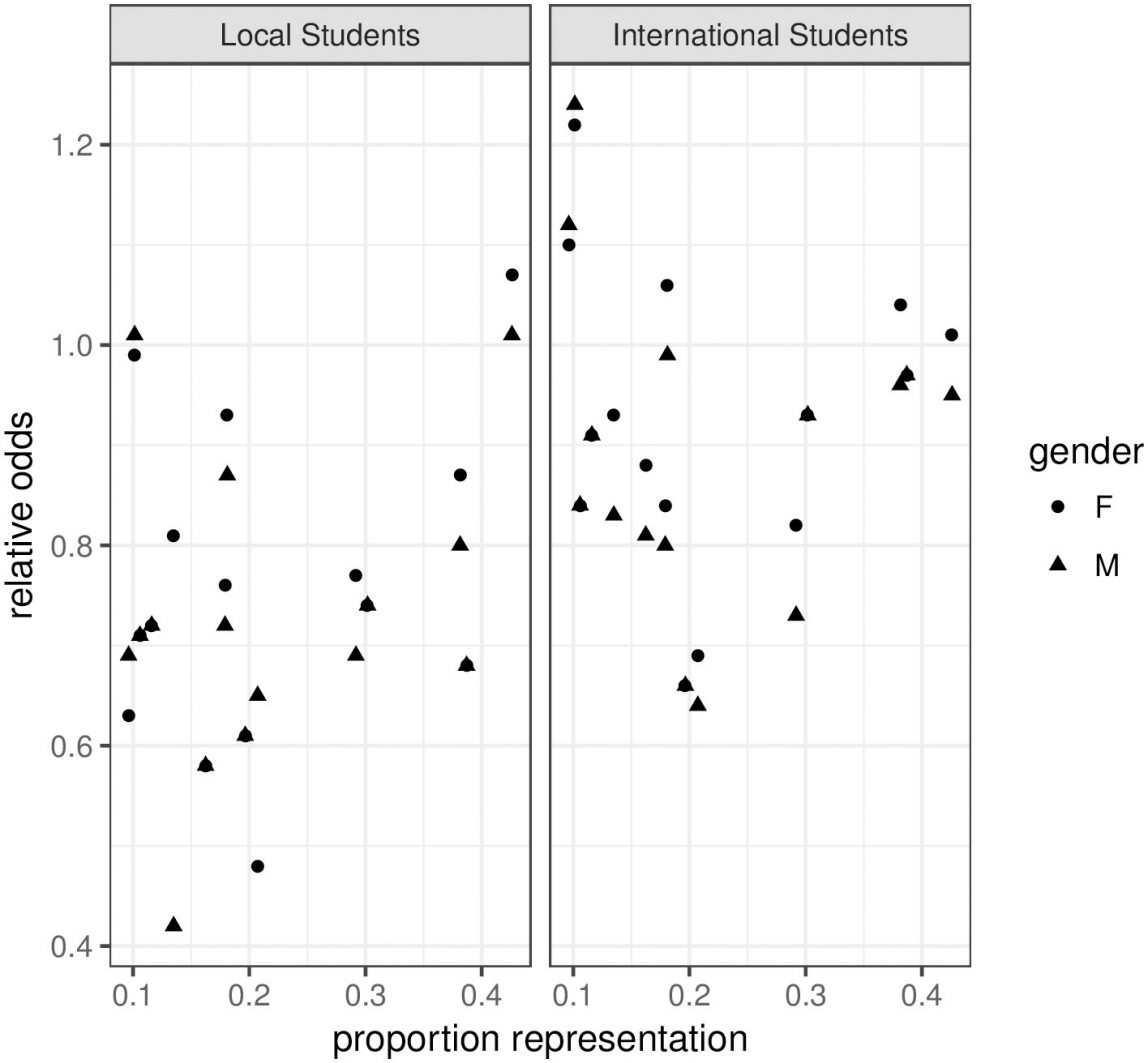
Plot of relative odds (y-axis) against the proportion of representation in the groups (female (E), female (NE), male (NE). Based on local student evaluations (left) and international student evaluations (right). Circles are from female students, triangles are from female students.

Fig 4 shows the relationship between proportional representation of the female (E = English speaking background /NE = non-English speaking background) and male (NE) teachers against the estimated relative odds or the size of the effect, values on the y-axis below the value 1 shows increasing size of negative effect. The proportions were calculated ignoring those for whom cultural background was missing. The left panel shows the result from local students, both male and female. Except for a point corresponding to female teachers with an English language background in the Engineering school, as the proportion representation increases, the estimated effect size approaches the value at 1, which indicates no effect. The graph suggests that there may be a relationship between staff representation and bias, giving a correlation of around 0.5. The extreme point in the top left hand corner corresponds to the women in the Engineering faculty who received better scores than the male teachers, which was somewhat unexpected. However, Fig 2 suggests that the relatively better performance by women is related to male teachers in Engineering who are not scoring well compared to male teachers in other faculties, as seen in the lower overall expected scores from Engineering.

The right panel of Fig 4 gives the relative odds from international students while, the relationship suggested by this plot is much weaker, this is perhaps not surprising given the complex interaction between culture of students and culture of teacher and gender.

### How influential are the biases?

**Fig 5:**
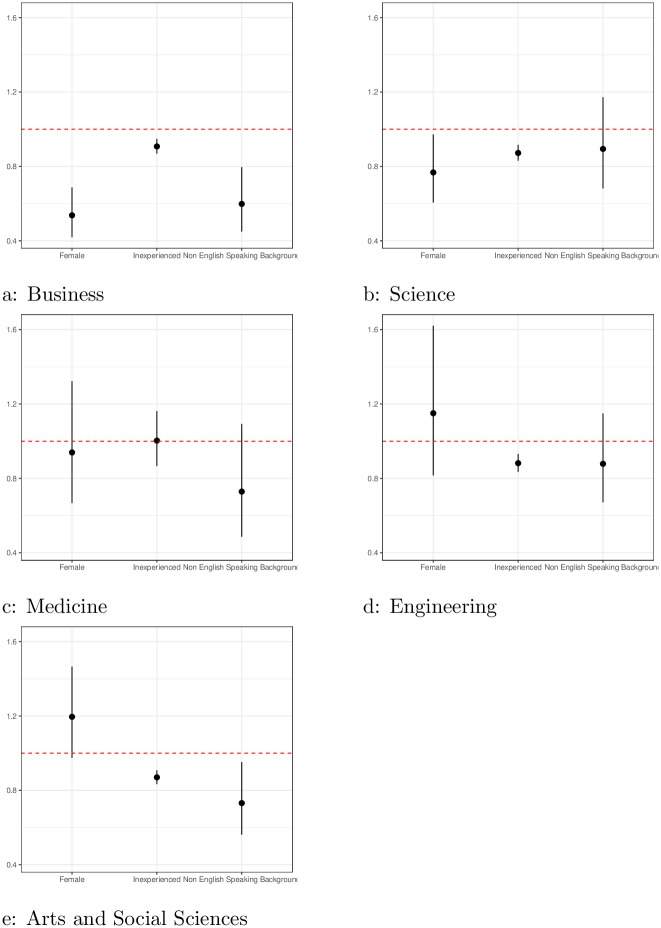
Gender effect from teaching evaluations. Points below the line at one indicate bias against groups (aggregated females, and non-English, teaching experience of all teachers and male and females of non-English speaking background) across male and female local and international students. Where solid lines do not intersect the line at 1, this indicates differences are statistically significant.

As mentioned above, many authors have questioned whether SET can really measure teaching effectiveness ([16]). While the definition of teaching effectiveness itself is a topic of debate, we consider here a slightly different question, that is, to what extent are SET scores driven by bias rather than teaching effectiveness. We use teaching experience as a proxy for teaching effectiveness, specifically using whether the teacher is teaching the course for the first time as a measure. While this is not a perfect proxy for teaching effectiveness, the University treats student feedback very seriously, and a low SET typically means the teacher will try much harder the next time s/he teaches. We find that the magnitudes of the biases in gender and culture are big. Fig 5 shows the effects of gender (pooled), cultural background (pooled) against a measure of teaching experience, i.e., teaching the course for the first time. We see here that the effect of gender or culture can outweigh the effect of teaching effectiveness, and in some faculties, such as Business, by quite a large margin.

### How good is the model?

We consider the model’s ability to correctly classify SET scores as an indicator for goodness of fit of the model. We use AUC (Area under the Receiver Operating Curve) for this purpose. The estimated parameters of the models have good ability to discriminate between scores being ≤ 5 versus 6, with AUC values ranging from 0.79-0.89, and excellent ability to discriminate between scores being ≤ 4 versus ≥ 5 (with AUC values ranging from 0.96 to 0.99). As the bulk of scores are between 5 and 6, it is expected that it will be harder to discriminate scores of 5 versus 6, than scores in the tail. Table 4 shows the AUC values for each model and cut-point.

**Table 4:** AUC values with 95% bootstrapped confidence intervals assess how well the model can discriminate SET scores ≤ 1, …, 5. Values between 0.7-0.8 are generally considered good, 0.8-0.9 is considered excellent whilst 0.9-1 is considered outstanding. The models are excellent at discriminating high SET scores (5-6) from low SET scores (≤ 4), with AUC’s between 0.96–0.99, and good at discriminating very high (6) from SET scores ≤ 5, with AUC’s 0.79-0.89.

	Bus	Sci	Med	Eng	Arts
<=1	0.96(0.95,0.97)	0.98(0.98,0.99)	0.98(0.97,0.99)	0.88(0.84,0.93)	0.95(0.94,0.97)
<=2	0.97(0.96,0.98)	0.99(0.98,0.99)	0.99(0.98,0.99)	0.92(0.9,0.95)	0.97(0.96,0.98)
<=3	0.99(0.98,0.99)	0.99(0.99,0.99)	0.99(0.99,0.99)	0.96(0.94,0.98)	0.98(0.98,0.99)
<=4	0.97(0.97,0.98)	0.98(0.97,0.99)	0.98(0.97,0.99)	0.96(0.96,0.97)	0.99(0.98,0.99)
<=5	0.82(0.8,0.85)	0.86(0.82,0.89)	0.81(0.77,0.85)	0.79(0.76,0.82)	0.89(0.88,0.9)

## Conclusion

This study analysed a large observational dataset of student evaluations of teaching, to detect potential bias, both in terms of gender and culture in student evaluation of teachers. Since surveys are voluntary, typical response rate is around 30% across the University, care should be taken when generalising these results to the more general student population. These results reflect the scoring patterns of those who responded. Note that when these surveys are used by the University administration, the effects of low response rates are not considered or accounted for. In the future, it would be interesting to study the effects of increasing survey response rate. In discarding teachers with missing culture information for the analyses involving culture, we have assumed that these information are missing at random. Although controlled experiments ([19]) are more ideal for studying a specific effect, they tend to suffer from small sample size, and can rarely address the complexity in the interplay between various factors that influence SET scores. When the sample sizes are large, such as the case with our study, then the findings of the observational study become more representative of a bigger population. With over 3,000 teachers in the sample, and over 44% of them female, and 38% with non-English background, the findings are less sensitive to individual specific traits.

Our findings suggest that SET scores are subject to different types of personal biases. To the best of our knowledge, this is the first study that has revealed statistically significant bias effects attributable to both gender and culture, and their interactions. We detected statistically significant bias against women and staff with non-English language backgrounds, although these effects do not appear in every faculty. Our findings on the effect of cultural background is novel and significant because in Australia, where the population is culturally diverse, current policy and administrative actions have focussed on addressing gender bias, but less on cultural or racial bias. We found some evidence that the proportion of women or staff with non-English language backgrounds in a faculty may be negatively correlated with bias, i.e., having a diverse teaching staff population may reduce bias. We also found that due to the magnitude of these potential biases, the SET scores are likely to be flawed as a measure of teaching performance. Finally, we found no evidence that student’s unconscious bias changes with the level of their degree program.

Throughout this paper, and in the title, we have used the term “bias” when describing the statistically significant effect females and non-English speaking teachers. It should be pointed out that one of the limitations of this study is that it is only able to show association, e.g., being female is associated with a lower SET score, we cannot say what really was the cause for a lower score. However, if SET is really measuring teaching quality, then the only plausible causes are either that females are generally bad teachers across a large population, or there’s bias, the same argument can be made for teachers who have non-English speaking background. Since we find no credible support that females, or someone with an accent, should generally be bad teachers, we have chosen to use the term “bias”. Comparing SET results from course evaluations where gender, or cultural background no longer shows up strong patterns, suggest that teaching evaluations may be evaluating the person, not the teaching effectiveness. Hence the effect we observe may be related to the student’s impression of the teacher in the context of the Australian university setting. Some evidence for this can be seen in the accompanying text responses where students comment on different aspects of the teacher, sometimes with a clearly gendered perspective, though this is beyond the scope of the present study.

Universities may be able to reduce bias in several ways, either by making sure they have staff diversity, by employing more under-represented staff in specific faculties, or through bias training for students. Making university students less biased may have enormous flow-on benefits for society, as university students represent a large proportion of future leaders in industry and government (for example all fortune 500 CEOs have at least a bachelor’s degree). The administration of the university on which our study is based, is proactively seeking change to minimise the effects of conscious and unconscious bias. Development of measures of teaching effectiveness which considers findings of this and other similar studies, would lead to enhanced teaching quality. A first step in this direction may be to consider bias correction to recalibrate the scores.

## Supporting information

S1 File Data underlying this study. (ZIP)
